# Supplementary material for: TBPL2/TFIIA complex establishes the maternal transcriptome through oocyte-specific promoter usage
Source: Nat Commun. 2020 Dec 22;11:6439. doi: 10.1038/s41467-020-20239-4 (PMC7755920; doi:10.1038/s41467-020-20239-4)
Supplement: Supplementary file 2 — Reporting Summary [file 41467_2020_20239_MOESM2_ESM.pdf]

nature research

Corresponding author(s): Laszlo Tora and Stephane D. Vincent

Last updated by author(s): 11/16/2020

Reporting Summary

Nature Research wishes to improve the reproducibility of the work that we publish. This form provides structure for consistency and transparency in reporting. For further information on Nature Research policies, see our Editorial Policies and the Editorial Policy Checklist.

Statistics

For all statistical analyses, confirm that the following items are present in the figure legend, table legend, main text, or Methods section.

n/a	Confirmed	
☐	☒	The exact sample size (*n*) for each experimental group/condition, given as a discrete number and unit of measurement
☐	☒	A statement on whether measurements were taken from distinct samples or whether the same sample was measured repeatedly
☐	☒	The statistical test(s) used AND whether they are one- or two-sided<br>*Only common tests should be described solely by name; describe more complex techniques in the Methods section.*
☒	☐	A description of all covariates tested
☒	☐	A description of any assumptions or corrections, such as tests of normality and adjustment for multiple comparisons
☐	☒	A full description of the statistical parameters including central tendency (e.g. means) or other basic estimates (e.g. regression coefficient) AND variation (e.g. standard deviation) or associated estimates of uncertainty (e.g. confidence intervals)
☐	☒	For null hypothesis testing, the test statistic (e.g. *F*, *t*, *r*) with confidence intervals, effect sizes, degrees of freedom and *P* value noted<br>*Give P values as exact values whenever suitable.*
☒	☐	For Bayesian analysis, information on the choice of priors and Markov chain Monte Carlo settings
☒	☐	For hierarchical and complex designs, identification of the appropriate level for tests and full reporting of outcomes
☐	☒	Estimates of effect sizes (e.g. Cohen's *d*, Pearson's *r*), indicating how they were calculated

Our web collection on statistics for biologists contains articles on many of the points above.

Software and code

Policy information about availability of computer code

Data collection	Mass spectrometry identification of proteins was performed using Proteome Discoverer (SequestHT and Percolator) version 1.4 (Thermo Fisher Scientific).
Data analysis	Data have been analysed using R 3.5.3 and the following R packages: DESeq2 version 1.22.2, CAGEr version 1.20.0, seqPattern version 1.14, SeqLogo version 1.48.0. Alignements were done using Bowtie1 version 1.2.2 (repeat elements analysis), Bowtie2 version 2.2.8 (SLIC-CAGE and RNA-seq analysis), or STAR version 2.7.0f (RNA-seq). Quantifications were done using htseq-count version 0.9.1 and annotations using HOMER version 4.10. Confocal images have been analysed using Fiji version 2.0.<br>All custom code is available upon request

For manuscripts utilizing custom algorithms or software that are central to the research but not yet described in published literature, software must be made available to editors and reviewers. We strongly encourage code deposition in a community repository (e.g. GitHub). See the Nature Research guidelines for submitting code & software for further information.

Data

Policy information about availability of data

All manuscripts must include a data availability statement. This statement should provide the following information, where applicable:

- Accession codes, unique identifiers, or web links for publicly available datasets
- A list of figures that have associated raw data
- A description of any restrictions on data availability

The datasets generated during the current study are available in different repositories:
proteomic data; ProteomeXchange PRIDE database (PXD0316347 [http://www.ebi.ac.uk/pride/archive/projects/PXD016347]) processed with a combined Mus musculus database generated using Uniprot [https://www.uniprot.org/uniprot/?query=proteome:UP000000589&sort=score] (Swissprot, release 2015_11, 16730

entries),
RNA-seq data; Gene Expression Omnibus database (GSE140090 [https://www.ncbi.nlm.nih.gov/geo/query/acc.cgi?acc= GSE140090]) processed with mm10 mouse genome assembly v96 [http://apr2019.archive.ensembl.org/index.html]
SLIC-CAGE data; ArrayExpress (E-MTAB-8866 [https://www.ebi.ac.uk/arrayexpress/experiments/E-MTAB-8866/]).) processed with mm10 mouse genome assembly v96 [http://apr2019.archive.ensembl.org/index.html].

Field-specific reporting

Please select the one below that is the best fit for your research. If you are not sure, read the appropriate sections before making your selection.

[x] Life sciences [ ] Behavioural & social sciences [ ] Ecological, evolutionary & environmental sciences

For a reference copy of the document with all sections, see nature.com/documents/nr-reporting-summary-flat.pdf

Life sciences study design

All studies must disclose on these points even when the disclosure is negative.

Sample size	No sample size calculation was performed due to the very limited amount of biological material (oocytes or young female ovaries): therefore all our data have been obtained from pools of independent preparations. The numbers of biological and/or technical replicates are indicated in the corresponding figure legends and in the Methods section, and they correspond to what is currently published in the field.
Data exclusions	No data exclusions were carried out.
Replication	Reproductibility of the results were replicated by generating independent datasets.<br>Proteomic analyses were performed independently twice, RNAseq data were performed once but with 3 independent biological replicates, SLIC-CAGE data were obtained from 2 biological WT replicates (that were pooled) and one mutant set of data.
Randomization	Randomization is not relevant as all the conditions were processed at the same time.
Blinding	Investigators were not blinded. Most of our data have been collected via computer analyses or were already quantitative (RT-qPCR) or only qualitative (IF, histology)

Reporting for specific materials, systems and methods

We require information from authors about some types of materials, experimental systems and methods used in many studies. Here, indicate whether each material, system or method listed is relevant to your study. If you are not sure if a list item applies to your research, read the appropriate section before selecting a response.

Materials & experimental systems

n/a	Involved in the study	
[ ]	[x]	Antibodies
[ ]	[x]	Eukaryotic cell lines
[x]	[ ]	Palaeontology and archaeology
[ ]	[x]	Animals and other organisms
[x]	[ ]	Human research participants
[x]	[ ]	Clinical data
[x]	[ ]	Dual use research of concern

Methods

n/a	Involved in the study	
[x]	[ ]	ChIP-seq
[x]	[ ]	Flow cytometry
[x]	[ ]	MRI-based neuroimaging

Antibodies

Antibodies used

Primary antibodies:
rabbit polyclonal anti-TBPL2 (3024): generated at IGBMC (this paper)
rabbit polyclonal anti-TAF7 (3475): generated at IGBMC (Bardot et al. , Development, 2017)
rabbit polyclonal anti-TFIIA-alpha: gift from HG Stunnenberg (Mitsiou and Stunnenberg, Mol Cell, 2000)

mouse monoclonal anti-TBPL2 (2B12): generated at IGBMC (Gazdag et al., Reproduction, 2007)
mouse monoclonal anti-TBP (3TF1-3G3): generated at IGBMC (Brou et al., EMBO J, 1993)
mouse monoclonal anti-TAF6 (25TA2G7): generated at IGBMC (Bell et al., Mol Cell, 2001)
mouse monoclonal anti-TAF10 (6TA2B11): generated at IGBMC (Mohan et al., Mol Cell Biol, 2003)
mouse monoclonal anti-GST (15TF21D10): generated at IGBMC (Nagy et al., Cell Mol Life Sci, 2010)

Secondary antibodies:
Alexa Fluor 488 goat anti-rabbit Ig (Invitrogen, #A-11108, lot 1829924)

	Peroxidase AffiniPure F(ab')$_2$ Fragment Goat Anti-Mouse IgG, Fcγ fragment specific (Jackson ImmunoResearch, #115-036-071, lot 132326)<br>Peroxidase AffiniPure Goat Anti-Rabbit IgG (H+L) (Jackson ImmunoResearch, #115-035-144, lot 132676)
Validation	Rabbit polyclonal anti-TAF7 (3475): raised against the whole human TAF7, also recognizes the mouse TAF7 (Bardot et al. , Development, 2017): WB, IP-WB, IP-MS, IF (this study);<br><br>rabbit polyclonal anti-TFIIA-alpha: raised against a human peptide, also recognizes the mouse TFIIA-alpha (Mitsiou and Stunnenberg, Mol Cell, 2000, Martianov et al., Science Report, 2016): WB<br><br>mouse monoclonal anti-TBPL2 (2B12): raised against a mouse specific peptide (Gazdag et al., Reproduction, 2007): WB and IF<br><br>mouse monoclonal anti-TBP (3TF1-3G3): raised against a human peptide, also recognizes the mouse TBP (Brou et al., EMBO J, 1993, Gazdag et al., Reproduction, Bardot et al., Development, 2017): WB, IF, IP-WB; IP-MS<br><br>mouse monoclonal anti-TAF6 (25TA2G7): raised against a human peptide, also recognizes the mouse TAF6 (Bell et al., Mol Cell, 2001, Bardot et al., Development 2017): WB<br><br>mouse monoclonal anti-TAF10 (6TA2B11): raised against a human peptide, also recognizes the mouse TAF10 (Mohan et al., Mol Cell Biol, 2003, Bardot et al., Development, 2017): WB, IF, IP-WB and IP-MS<br><br>mouse monoclonal anti-GST (15TF21D10): raised against a peptide, validated as a non specific antibody for mock (Nagy et al., Cell Mol Life Sci, 2010; Bardot et al., Development, 2017): IP-MS<br><br>The new 3024 anti-TBPL2 polyclonal antibody was raised against a mouse specific TBPL2 peptide : it has been validated by immunoprecipitation followed by western blot from cells overexpressing or not TBPL2 (that is only expressed in oocytes) in the current study (Supplementary Figure 1b).<br><br>IP: immunoprecipitation, WB: western blot, IF: immunofluorescence, MS: mass spectrometry

Eukaryotic cell lines

Policy information about cell lines

Cell line source(s)	The NIH3T3-II10 and NIH3T3-K2 have been created in our lab and have been published (Gazdag et al 2007, Reproduction).
Authentication	The authentification was carried out by western blot analyses of mTBPL2 expression (see also Supplementary Figure 1b in the current study).
Mycoplasma contamination	All cell lines were negative for mycoplasma contamination.
Commonly misidentified lines (See ICLAC register)	no commonly misidentified cell lines were used in the study.

Animals and other organisms

Policy information about studies involving animals; ARRIVE guidelines recommended for reporting animal research

Laboratory animals	Two month old female rabbits, White New-Zealand strain (2 to 2.5 kg) were used to generate the anti-TBPL2 antibodies.<br><br>The Tg(Zp3-Cre), Taf7flox and Tbpl2 KO mouse lines have already been described (see references in the Methods section). 8-12 weeks old males and females were used for breeding and post natal days 7 or 14 females were used for transcriptomics and proteomics experiments. For histology and immunofluorescence 6-8 weeks old females were used, and for super ovulation 4 weeks old females were used.<br><br>All animals were housed under a controlled temperature (22°C, +/- 1°C) , humidity (55 ± 5%) and a 12 light-dark cycle (lights on at 7 AM) with free access to food and water.
Wild animals	no wild animals were used in the study.
Field-collected samples	no field-collected samples were used in the study.
Ethics oversight	Animal experimentations were carried out according to animal welfare regulations and guidelines of the French Ministry of Agriculture and procedures were approved by the French Ministry for Higher Education and Research ethical committee C2EA-17 (project n°2018031209153651).

Note that full information on the approval of the study protocol must also be provided in the manuscript.
