# Supplementary material for: TBPL2/TFIIA complex establishes the maternal transcriptome through oocyte-specific promoter usage
Source: Nat Commun. 2020 Dec 22;11:6439. doi: 10.1038/s41467-020-20239-4 (PMC7755920; doi:10.1038/s41467-020-20239-4)
Supplement: Supplementary file 3 — Description of Additional Supplementary Files [file 41467_2020_20239_MOESM3_ESM.pdf]

Description of Additional Supplementary Files

Supplementary Data 1: anti TBPL2 immunoprecipitation coupled with mass spectrometry from ovary whole cell extracts (FDR5%, 1 peptide per protein);

Supplementary Data 2: anti TBP immunoprecipitation coupled with mass spectrometry from ovary whole cell extracts (FDR5%, 1 peptide per protein);

Supplementary Data 3: anti TAF7, anti TAF10 and anti TBPL2 sequential immunoprecipitation coupled with mass spectrometry from ovary whole cell extracts (FDR5%, 1 peptide per protein);

Supplementary Data 4: anti TBPL2 immunoprecipitation coupled with mass spectrometry from gel filtration fraction (FDR5%, 1 peptide per protein);

Supplementary Data 5: Differential gene expression analysis from post-natal (P) 7 and P14 oocytes (wild type vs *Tbpl2*$^{-/-}$);

Supplementary Data 6: Gene ontology analyses using PANTHER from RNA seq data;

Supplementary Data 7: Gene ontology analyses using PANTHER from SLIC CAGE data (down regulated promoters TSS cluster).
